# 2419: Clinical research management and regulatory compliance: A graduate distance learning model

**DOI:** 10.1017/cts.2017.179

**Published:** 2018-05-10

**Authors:** Alberto M. Carrera, Lizbelle De Jesús-Ojeda, Estela Estape, Lourdes E. Soto de Laurido

## Abstract

OBJECTIVES/SPECIFIC AIMS: Goal—broaden the academic offer to enhance clinical and translational research productivity and cost effectiveness. Objective—implement a distance learning program on conducting proficient research management. METHODS/STUDY POPULATION: Needs assessment attested students’ interest in enrolling and willingness to recruit graduates by the research industry and academia. A master of science in clinical research management and regulatory compliance (MS-CRMRC) was developed using the Core Competency Domains for Clinical Research Professional. Experts from research academia, pharmaceutical industry, composed a Proposal Development Committee. RESULTS/ANTICIPATED RESULTS: Access of a distance learning MS-CRMRC program for students with time constrains. Competent research professional graduates working side by side with the principal investigator on onsite teamwork management, to streamline research processes in compliance to regulations. DISCUSSION/SIGNIFICANCE OF IMPACT: Improvement of clinical and translational research productivity and efficient use of grants funds prevails as a generalized concern. The MS-CRMRC offers an accessible alternative to empower the research enterprise by developing knowledgeable skilled professionals to tackle this need.

